# Evaluation of the competence of an artificial intelligence-assisted colonoscopy system in clinical practice: A *post hoc* analysis

**DOI:** 10.3389/fmed.2023.1158574

**Published:** 2023-04-06

**Authors:** Wei Zuo, Yongyu Dai, Xiumei Huang, Ren-qun Peng, Xinghui Li, Hao Liu

**Affiliations:** Department of Gastroenterology, Chongqing Rongchang District People's Hospital, Chongqing, China

**Keywords:** artificial intelligence, coloscopy, colon polyps, adenoma, polyps size measurement

## Abstract

**Background:**

Artificial intelligence-assisted colonoscopy (AIAC) has been proposed and validated in recent years, but the effectiveness of clinic application remains unclear since it was only validated in some clinical trials rather than normal conditions. In addition, previous clinical trials were mostly concerned with colorectal polyp identification, while fewer studies are focusing on adenoma identification and polyps size measurement. In this study, we validated the effectiveness of AIAC in the clinical environment and further investigated its capacity for adenoma identification and polyps size measurement.

**Methods:**

The information of 174 continued patients who went for coloscopy in Chongqing Rongchang District People’s hospital with detected colon polyps was retrospectively collected, and their coloscopy images were divided into three validation datasets, polyps dataset, polyps/adenomas dataset (all containing narrow band image, NBI images), and polyp size measurement dataset (images with biopsy forceps and polyps) to assess the competence of the artificial intelligence system, and compare its diagnostic ability with endoscopists with different experiences.

**Results:**

A total of 174 patients were included, and the sensitivity of the colorectal polyp recognition model was 99.40%, the accuracy of the colorectal adenoma diagnostic model was 93.06%, which was higher than that of endoscopists, and the mean absolute error of the polyp size measurement model was 0.62 mm and the mean relative error was 10.89%, which was lower than that of endoscopists.

**Conclusion:**

Artificial intelligence-assisted model demonstrated higher competence compared with endoscopists and stable diagnosis ability in clinical use.

## Introduction

Colonoscopy is currently the first-line approach to detect colorectal adenomas and tumors. Standardized colonoscopy screening can effectively reduce the incidence and mortality of colon cancer (1). Polyp detection rate and adenoma detection rate are both important clinical quality indicators of colonoscopy, which are heavily dependent on the heterogeneity of the competence and qualification of endoscopists (2). A previous meta-analysis showed that about 22–26% of colon adenomas were missed during colonoscopy (3). All the missed lesions could further develop into colon cancers, which constituted approximately 60% of interval colorectal cancers after colonoscopy (4).

In recent years, artificial intelligence (AI) based on deep learning has been widely applied in endoscopy to assist endoscopists from various perspectives (5). It has played a significant role in monitoring the blind spots of esophagogastroduodenoscopy, quality control of colonoscopy, and endoscopic diagnosis of early gastric cancer (6–9). In addition to the above application, artificial intelligence-assisted coloscopy (AIAC) systems to automatically detect colorectal polyps and recognize colorectal adenoma have also been developed (7, 10) and validated in several clinical trials and were approved that the AIAC could decrease the miss rate of colorectal polyps and adenomas (8). A recent meta-analysis concluded that the use of AIAC systems was effective in increasing the detection rate of colorectal polyps and adenomas by approximately 10% (6). Another study has achieved real-time measurement of polyp size based on deep learning during colonoscopy to assist physicians in selecting polyp treatment strategies (8).

Although AIAC systems have been validated in some image datasets, video datasets, man–machine competitions, and some prospective randomized controlled trials, their efficacy is still unclear when they are applied in the real clinical environment. Considering the more stringent requirements of clinical trials compared to common clinical practice, the effectiveness of AIAC systems in the clinical environment needs to be further validated. Our healthcare center started using the AIAC system for colonoscopy in 2020, and in this study, we retrospectively collected consecutive coloscopy cases for statistical analysis to explore the performance of AIAC in the clinic in three aspects, including colorectal polyp detection, colorectal adenoma diagnosis and polyp size measurement.

## Methods

### AIAC system

The AIAC system applied in our healthcare center was constructed based on deep learning. Google’s Keras 2.1.5 based on TensorFlow 1.12.2 deep learning framework was used to train the AIAC. Early stopping was used to watch a validation curve and stop training when the validation loss did not decrease 10 times during the training process. The system mainly contains the following three parts.

1. Polyp recognition model: The model was constructed to detect the colorectal polyps and it was based on the Yolo V3 network, using 19,583 colonoscopy images as the training dataset. In the test dataset which included 1997 images, its sensitivity was 96.65%, and specificity was 91.75%. More detailed information about the train and test of the polyp recognition model was described in the previous study (11).
2. Colorectal adenoma recognition model: Based on the Res-net 50 network, 2,699 narrow band imaging (NBI) non-magnified images of colorectal adenoma polyps and 1846 non-adenomatous polyps were used to train and validate the colorectal adenoma recognition model to distinguish the colorectal adenoma from hyperplastic polyps automatically. The accuracy of the model was as high as 90.16%. More detailed information about the train and test of the colorectal adenoma recognition model was described in a previous study (10).
3. Colorectal polyp size measurement model: The model was developed based on the U-net++ network to segment the biopsy forceps, which was trained using 4,835 images that contained biopsy forceps. The average intersection over union (IoU) ratio of biopsy forceps segmentation was 0.92. The model could calculate the polyp size at the pixel level by predicting the minimum external rectangular box length of the polyp which was detected by the polyp recognition model. The segmented biopsy forceps and the known forceps diameter (in millimeter level) when the biopsy forceps were opened can be used as the scale bar to convert the pixel unit of minimum external rectangular box length into millimeters. In a previous study, the mean absolute error of the model for measuring polyps was 0.24 mm and the mean relative error was 9.74%. More detailed information about the train and test of the colorectal polyp size measurement model was described in the previous study (12).

### Study design and data collection

This *post hoc* analysis study was conducted in Chongqing Rongchang District People’s Hospital from September 1st, 2020 to June 30th, 2021. The information of 174 continued patients who went for coloscopy with detected colon polyps was retrospectively collected, including colonoscopy white light images and NBI images, baseline information of patients, endoscopic diagnosis, and pathological diagnosis. The images and videos used in this study were generated by two endoscope brands (*Olympus CV-290* and *Pentax EPK-i7000* from Japan). Then, their coloscopy images were divided into three validation datasets, that is, polyp dataset, polyp/adenoma dataset (all containing NBI images), and polyp size measurement dataset (videos which contain biopsy forceps and polyps) to assess the competence of the artificial intelligence system, and compare its diagnostic ability with endoscopists with different experiences. The study was approved by the Ethics Committee of Chongqing Rongchang District People’s Hospital and Informed consent was waived since it was a retrospective study.

### Evaluation of the AIAC system and comparing it with endoscopists

To further validate the AIAC system in the clinical environment, we tested the system using the three aforementioned datasets and compared the diagnostic accuracy of the AIAC system with endoscopists with difference experiences.

1. Validation of the polyp recognition model: We calculated the sensitivity of the model to represent the competence of AIAC in polyp detection by the polyps dataset. Subgroup analysis was conducted according to the polyp location and size. The model was validated on the basis of the total number of polyps and the number of polyps images, respectively. The formula was: sensitivity of polyp recognition = true positive/ (true positive + false negative).
2. Validation of the colorectal adenoma recognition model and comparing its performance with endoscopists: Taking the pathology diagnosis as the gold standard, the accuracy of the colorectal adenoma recognition model in the polyp/adenoma dataset was calculated. The diagnostic accuracy of the model was calculated by the formula: diagnostic accuracy of adenoma = (true positive + true negative)/overall. In addition, three endoscopists with different years of coloscopy experience (two of them are endoscopists with more than 3 years of experience in colonoscopy and one are senior endoscopist with more than 5 years of experience in colonoscopy) were invited to participate in the man–machine competition to compare the competence of colorectal adenoma recognition of the model with endoscopists.
3. Validation of the colorectal polyp size measurement model and comparing its performance with endoscopists: Taking pathological findings as the gold standard, the mean absolute error of the colorectal polyp size measurement model in the polyp size measurement dataset was calculated. In addition, three endoscopists with different years of coloscopy experience (two of them are endoscopists with more than 3 years of experience in colonoscopy and one are senior endoscopist with more than 5 years of experience in colonoscopy) were invited to participate in the man–machine competition to compare the competence of colorectal polyp size measurement of the model with endoscopists.

### Statistical analysis

Sensitivity and accuracy were calculated to evaluate the polyp recognition performance and adenoma recognition performance of the AIAC system. Mean absolute error and mean relative error were calculated to evaluate the polyp size measurement performance of the AIAC system. In the man–machine competition, the difference in diagnostic accuracy between endoscopists and the adenoma recognition model was compared using McNemar’s test, and the difference in mean absolute error and mean relative error of polyp size measurement between endoscopists and the polyp size measurement model was compared using paired-sample Student’s t test, with *p* < 0.05 indicating that there was the statistical difference.

## Results

### Patient enrollment and baseline information

From September 1st, 2020 to June 30th, 2021, information on patients who went for coloscopy in Chongqing Rongchang District People’s hospital was collected in the study, and those who failed to complete the coloscopy, younger than 18 years old, or those who were at that time pregnant or lactating were excluded. 174 patients were included in the study with a mean age of 51.14 years, of which 143 patients were examined with an Olympus endoscopy system and 31 patients were examined with a Pentax endoscopy system as shown in Table 1. One hundred and eighty polyp lesions were detected in the above patients, of which 6 had two or more polyp lesions. A total of 62 videos were collected for polyp size measurement.

**Table 1:** Baseline information of included patients.

Items		Results
Age(year, mean ± SD)		51.14 ± 13.61
Gender (male/female, %)		(94/80) 54.02%
Endoscopy brands		
	Number of Pentax	31 (17.82%)
	Number of Olympus	143 (82.18%)
Polyp location		
	Cecum-ascending colon	56 (32.18%)
	Transverse colon	40 (22.99%)
	descending colon	40 (22.99%)
S	Sigmoid-rectum	44 (25.29%)
Polyp size		
	≤5 mm	103 (59.20%)
	6–9 mm	36 (20.69%)
	≥10 mm	41 (23.56%)

#SD, standard deviation.

### Baseline information of the clinical validation datasets

**Figure 1:**
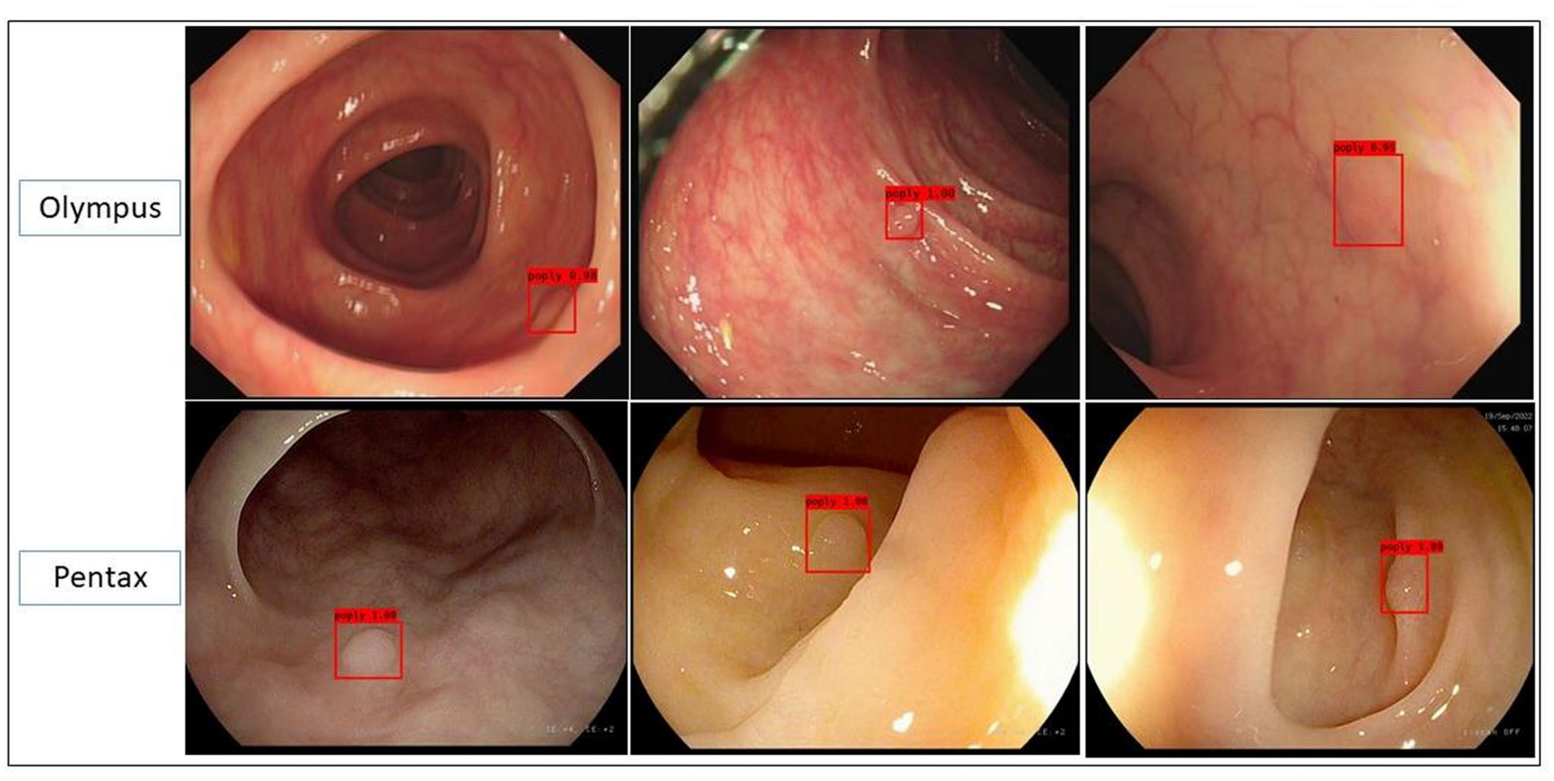
Representative images predicted by the polyp recognition model in the polyp dataset in both Olympus and Pentax brands.

Three clinical validation test datasets were successfully constructed based on the above data from 174 patients. The polyps dataset contained 180 polyp lesions from 174 patients, with a total of 707 polyp images; the polyps/adenomas dataset contained 64 polyp lesions and 80 adenoma lesions from 143 patients (all of them contained NBI images from Olympus); the polyp size measurement dataset (video dataset) contained 62 videos from patients who had polyps and in the videos, biopsy forceps were used. Those videos contained 37 videos from Olympus and 25 videos from Pentax (Figure 1; Table 2).

**Table 2:** Basic information of clinical validation dataset.

Dataset	Number of patients	Endoscopy brands	Number of lesions
Polyp dataset	174	Olympus (143), Pentax (31)	180 Polyps
polyps/adenomas dataset (NBI images)	143	Olympus (143)	64 Hyperplastic polyps; 80 adenoma polyps
polyp size measurement dataset	62	Olympus (37), Pentax (25)	62 Polyps

### Competence of AIAC system and comparison with endoscopists

#### Sensitivity of polyp recognition model

**Figure 2:**
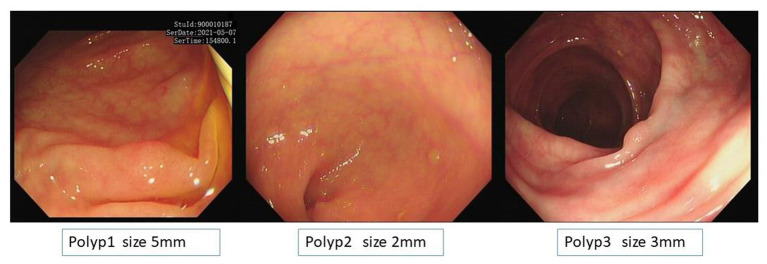
Images of the three unsuccessful recognition polyps and polyp 1 is the unsuccessful false-negative polyp when it was calculated based on image basis.

Among 180 polyps in 174 patients, the polyp recognition model successfully detected 177 polyps with a sensitivity of 99.40% on the basis of polyps and 99.58% on the basis of polyps images. The model worked well in subgroup analysis when it came to different endoscopy brands, polyp locations, or sizes. The model demonstrated higher competence in medium to large polyps compared to polyps ≤5 mm, as shown in Table 3. In the dataset, there were three images that contained three different polyps were failed to be diagnosed by the model, and the three images were shown in Figure 2. Among the three polyps, two of them were successfully recognized by the model in other images of the polyps, but only one polyp failed to be recognized in all the images of it.

**Table 3:** Sensitivity of polyp recognition and subgroup analysis.

Classification		Polyp number	Sensitivity (patient)	Sensitivity (image)
Overall polyps		180	(179/180) 99.40%	(704/707) 99.58%
Endoscopy brands				
	Olympus	145	(144/145) 99.31%	(569/572) 99.48%
	Pentax	35	(35/35) 100%	(135/135)100%
Polyp location				
	ascending colon	56	(56/56)100%	(217/217) 100%
	Transverse colon	40	(40/40)100%	(158/159) 99.37%
	descending colon	40	(40/40)100%	(163/163) 100%
	Sigmoid colon and rectum	44	(43/44) 97.73%	(166/168) 98.81%
Polyp size				
	≤5 mm	103	(102/103) 99.03%	(412/415) 99.28%
	6–9 mm	36	(36/36) 100%	(140/140) 100%
	≥10 mm	41	(41/41) 100%	(152/152) 100%

#### Accuracy of colorectal adenoma recognition model and comparison with endoscopists

**Figure 3:**
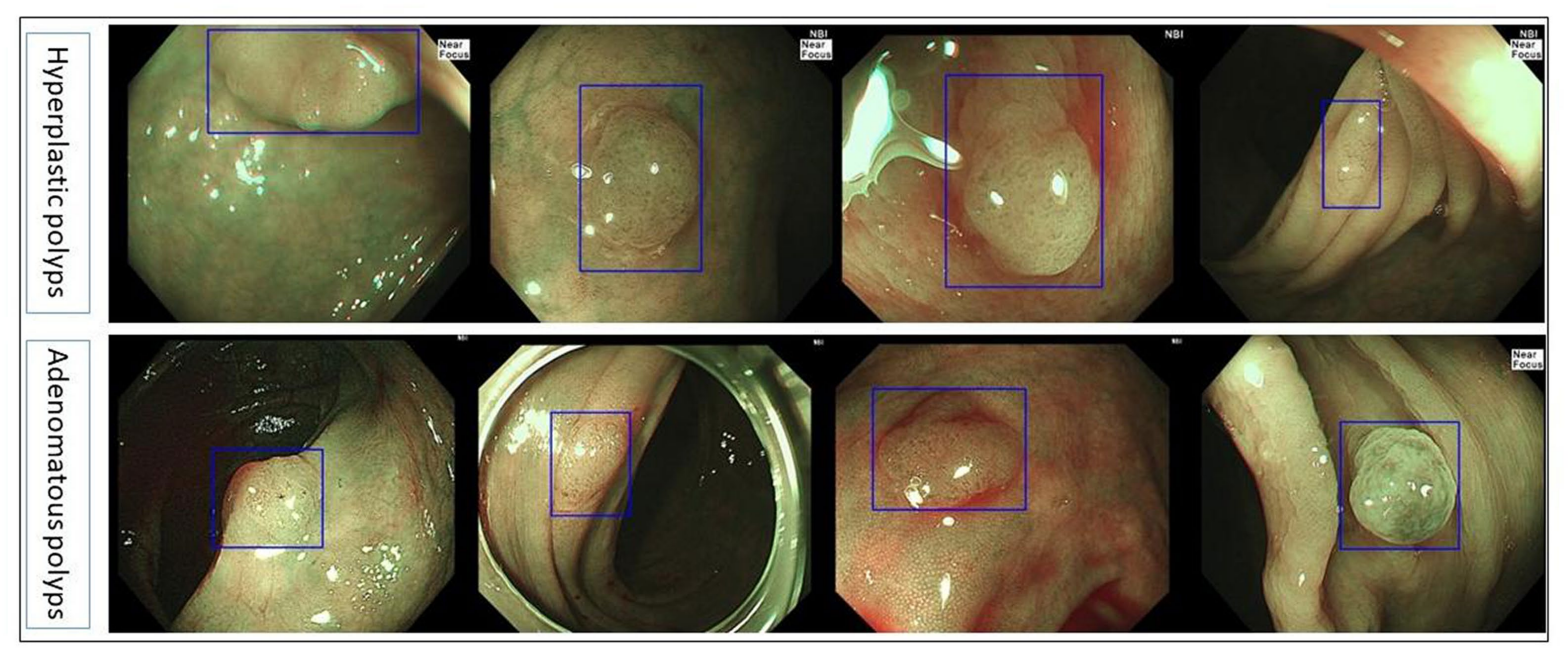
Typical images of misdiagnosed colorectal adenoma by the colorectal adenoma recognition model.

**Figure 4:**
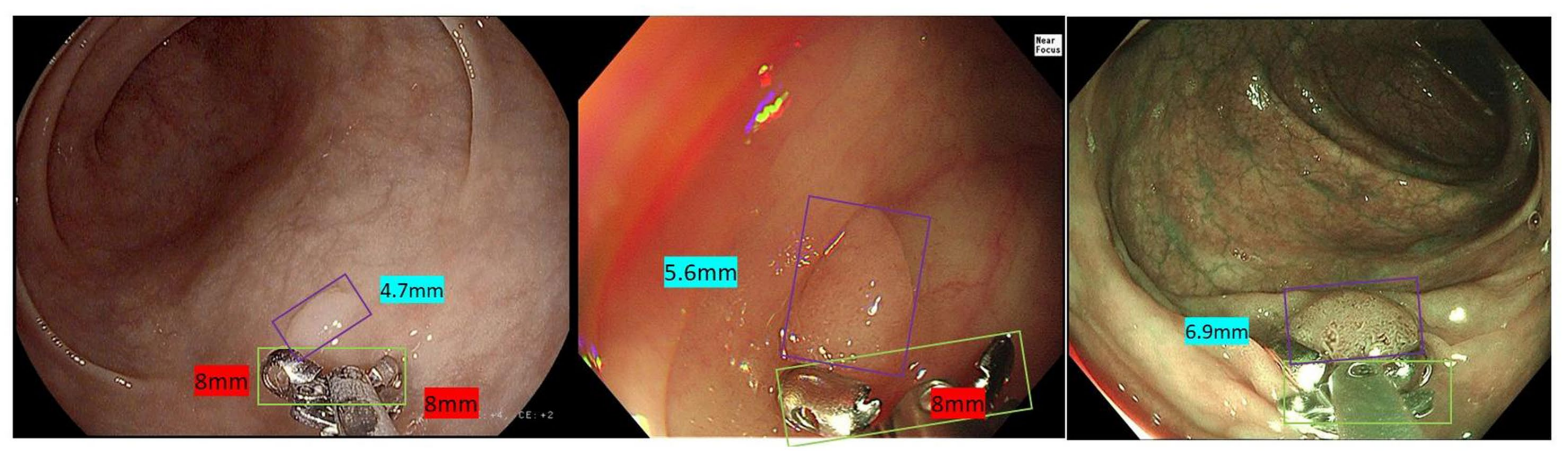
Automatic measurement process of the polyp size measurement model.

The collected NBI images from Olympus endoscopy which contained polyps were used as a polyp/adenoma dataset to test the accuracy of the colorectal adenoma recognition model and compare the accuracy of the model and endoscopists. The sensitivity, specificity, and accuracy of the colorectal adenoma recognition model were 92.5, 93.75, and 93.06%, respectively, which were significantly higher than all three endoscopists (*p* < 0.0001). More details are shown in Table 4. Figure 3 demonstrates some of the typical images of misidentified colorectal adenoma predicted by the model (Figure 4).

**Table 4:** Comparison of differences in diagnostic ability of colorectal adenoma between endoscopists with different levels and the colorectal adenoma recognition model.

	Endoscopists 1	Endoscopists 2	Senior endoscopists 3	Model
Sensitivity	88.75%	90.00%	91.25%	92.50%
Specificity	73.44%	75.00%	78.13%	93.75%
Accuracy	81.94%	83.33%	85.42%	93.06%

#### Accuracy of colorectal polyp size measurement model and comparison with endoscopists

Taking the size of pathologically resected colorectal polyps as the gold standard, the mean absolute error of the colorectal polyp size measurement model was 0.62 mm and the mean relative error was 10.89% in the collected polyp size measurement video dataset. It was significantly lower than that of endoscopists (mean absolute error *p* < 0.0001; mean relative error *p* < 0.0001). To sum up, their polyp size measurement model showed higher competence in the clinical environment. The details are shown in Table 5.

**Table 5:** Comparison of the difference of polyp size measurement competence in colorectal polyp size measurement model with endoscopists.

	Endoscopists 1	Endoscopists 2	Senior endoscopists 3	Model	*p*-Value
Mean absolute error	1.74 mm	1.69 mm	1.65 mm	0.62 mm	< 0.0001
Mean relative error	31.50%	31.80%	30.81%	10.89%	< 0.0001

## Discussion

In this study, we retrospectively collected consecutive colonoscopies combined with clinicopathological information from 174 patients in Chongqing Rongchang District People’s Hospital from September 1st, 2020 to June 30th, 2021, and constructed three validation datasets, polyp dataset, polyp/adenoma dataset and polyp size measurement dataset to validate the AIAC system in clinical practice, which composed polyp recognition model, colorectal adenoma recognition model and colorectal polyp size measurement model. The AIAC system could accurately identify colorectal polyps with a sensitivity of 99.58%, and diagnose colorectal adenoma with an accuracy of 93.06%, which was significantly higher than that of the endoscopists. In addition, the AIAC system could achieve automatic measurement of polyp size, and the mean absolute error and mean relative error of polyp size measurement were significantly lower than those of endoscopists.

Colonoscopy is currently the first-line approach for diagnosing colon diseases, and it has been recommended for colorectal cancer screening in many western countries which was approved to be cost-effective (13). However, as a highly technique-dependent operation that requires endoscopists’ operating skills and diagnostic ability, the quality of colonoscopy is greatly influenced by the endoscopist’s competence and fatigue of work (14). With the invention of deep learning and deep convolutional neural network in 2015 (15), artificial intelligence has made a tremendous leap in image recognition and has been widely used in the field of gastrointestinal endoscopy (16). Among the various applications, colorectal polyp recognition and colorectal adenoma diagnosis have been sufficiently studied (17–19), and several AI-assisted systems have been used in hospitals. Previous studies have reported that the application of artificial intelligence-assisted coloscopy systems can effectively increase the polyp detection rate and adenoma detection rate by approximately 10% (6). The system showed higher assistance ability especially and could eliminate the impact of high workloads on endoscopists (20). However, these studies mostly are observational clinical studies or randomized controlled trials which usually followed strict inclusion and exclusion criteria. In the universal colonoscopy procedure, the clinical environment is complex, and the endoscopic diagnosis is usually affected by many interfering factors such as bowel preparation, cooperation of patients, and so on. The diagnostic competence of AI models in clinical practice may differ from studies of previous trials, but it still has not been investigated.

The present study included consecutive cases of successful colonoscopies to fully validate the AIAC system in clinical practice without any interference. The AIAC system was constructed based on deep learning and then updated several versions. Some algorithms were added to modify the system like spot repair, which optimized the models and improved the accuracy of the models by 3–10% compared to our pervious study.174 patients were included and a total of 180 colon polyps were detected, of which, only one polyp was not successfully identified by the AIAC system. In all the test datasets, only three images that contained three polyps were misdiagnosed by the model and two of them were successfully recognized by the model in other images of the polyps, thus the 2 polyps were not missed. Only one polyp failed to be recognized in all the images of it. In the polyp/adenoma dataset, we only included NBI images from Olympus. Narrow-band imaging international colorectal endoscopic classification (NICE) was adopted to classify the polyps (21–23). More specifically, polyps of NICE type I were classified into hyperplastic polyps while polyps of NICE type II or above should be identified as adenoma lesions (20). The accuracy of the colorectal adenoma recognition model was 93.06%, which was significantly higher than that of previous studies, using pathological diagnosis as the gold standard, from which we can reach the conclusion that the AIAC system worked well on colorectal polyp recognition and adenoma diagnosis in clinical practice.

In addition, the diameter of the opened biopsy forceps was chosen as the scale to assist the measurement of polyps in the present study. The size of lesions is proven to be closely related to the prognosis of patients and also an important indicator for treatment strategy. As a result, it was required that the size of polyp lesion should be accurately reported on endoscopy reports (24). Several previous studies have proposed some methods for polyp measurement, including dipsticks, aligning tools and so on (25). However, these methods are cumbersome and inaccurate so they have not been used in clinical practice. At present, endoscopists only make a rough estimation of lesion size based on their experience. The polyp size measurement model validated in this study provides a convenient and operable solution to this problem. The model showed lower estimation errors and it was more reliable on polyp measurement in the test dataset compared to endoscopists. Besides, it achieved automatic measurement with out *in vitro* measurement work. In clinical practice, the images of endoscopy used to be distorted and the shape of polyps is changed which may contributes to the misestimated polyp size by endoscopists. However, the AIAC system used the biopsy forceps as the scale to estimate the polyp size, which may eliminate the interference of images distortion caused by the endoscopy. It’s also helpful to estimate the accurate size of the lesion since it measures the lesion *in vivo*. The resected lesion may be shrunken and the *in vitro* measurement will have more distortion when compared to the *in vivo* measurement.

The present study has some significant strengths. Firstly, we investigated the competence of the AIAC system in clinical practical use. Statistics were conducted based on continuous clinical datasets without strict inclusion and exclusion criteria, of which the conclusions are more reliable and closer to real clinical feedback. The conclusion could provide a more realistic basis for the development of relevant guidelines, standards and policies. Secondly, we validated a total of three models and analyzed the clinical effectiveness of three models for colorectal polyp detection, adenoma diagnosis and polyp size measurement, which could basically cover most of the needs of colonoscopy in terms of application scenarios in clinical use. Finally, in addition to the Olympus colonoscopy system which is the mainstream brand of the market, we also verified the effectiveness of this system in Pentax colonoscopy. The AIAC system demonstrated a similar ability of polyp recognition to that of Olympus, which further proved the robustness and universality of the system. The system could meet a variety of clinical use needs with high compatibility. Of course, there are some limitations to this study. Another commonly used endoscope, the Fuji endoscopy, was not analyzed in this study, and we need to further expand the validation in more endoscopy brands in the future. Secondly, the biopsy forceps was chosen as the scale to measure the polyp size since it is the most frequently used accessories during endoscopy. However, it means the measurement function of AIAC cannot work without biopsy forceps. We need to adopt more kinds of accessories to fulfill the clinical needs. Thirdly, clinical importance of colorectal polyps is not determined by only polyp size but also pit pattern, surface type and so on. More features of colorectal polyps should be combined by the AIAC system to better recognize the polyps. Lastly, in the study, only three endoscopists were successfully invited to participated in the man–machine competition. We pretty agreed that more endoscopists should be included in the future to make the conclusion more convincing. And we illustrated the point in the limitation.

In summary, this study validated the AIAC system in aspects of colorectal polyp recognition, colorectal adenoma diagnosis, and colorectal polyp size measurement in clinical practice, and the above models worked stably in the complex clinical environment.

## Data availability statement

The original contributions presented in the study are included in the article/supplementary material, further inquiries can be directed to the corresponding author.

## Ethics statement

The studies involving human participants were reviewed and approved by Ethics Committee of Chongqing Rongchang District People’s Hospital. Written informed consent for participation was not required for this study in accordance with the national legislation and the institutional requirements.

## Author contributions

HL and WZ designed the study and completed the manuscript. YD, XH, and R-qP participated the study in man–machine competition, and XL revised the manuscript. All authors contributed to the article and approved the submitted version.

## Funding

This work was supported by Chongqing science commission and healthcare commission union medical research project, 2021MSXM182, to HL; Early digestive cancer doctors’ training project of national cancer early diagnosis and treatment program (rural area) expert committee in 2022, GTCZ-2022-CQ-55-0004; and Early digestive cancer doctors’ training project of national cancer early diagnosis and treatment program (rural area) expert committee in 2022, GTCZ-2022-CQ-55-0009.

## Conflict of interest

The authors declare that the research was conducted in the absence of any commercial or financial relationships that could be construed as a potential conflict of interest.

## Publisher’s note

All claims expressed in this article are solely those of the authors and do not necessarily represent those of their affiliated organizations, or those of the publisher, the editors and the reviewers. Any product that may be evaluated in this article, or claim that may be made by its manufacturer, is not guaranteed or endorsed by the publisher.
